# Changing Pattern of Visceral Leishmaniasis, United Kingdom, 1985-2004

**DOI:** 10.3201/eid1208.050486

**Published:** 2006-08

**Authors:** Aeesha N.J. Malik, Lawrence John, Anthony D.M. Bryceson, Diana N.J. Lockwood

**Affiliations:** * Hospital for Tropical Diseases, London, United Kingdom

**Keywords:** Visceral leishmaniasis, HIV, imported disease, tourist, sodium stibogluconate, liposomal amphotericin, leishmania serology

## Abstract

A 20-year (1985–2004) retrospective review of 39 patients with imported visceral leishmaniasis found that tourism to Mediterranean countries and HIV infection were associated with visceral leishmaniasis. Diagnosis was often delayed. Treatment with liposomal amphotericin B has improved prognosis. Visceral leishmaniasis should be made a reportable disease.

Each year, 500,000 new cases of visceral leishmaniasis (VL) are reported worldwide. The number of cases and endemic foci for VL have increased during the past 2 decades (*1**–**4*). These increases may be the result of improved detection and surveillance methods, or they may be actual increases in numbers, possibly driven by increasing rates of HIV infection (*5**,**6*). VL is a well-recognized but uncommon imported disease in the United Kingdom, but it is not reportable, so information on its importation is incomplete. Data for this report (e.g., date of confirmation of infection, country where acquired, HIV status) were collated and supplied by the Travel Health Surveillance Section of the Health Protection Agency, Communicable Disease and Surveillance Centre, United Kingdom.

## The Study

**Figure 1:**
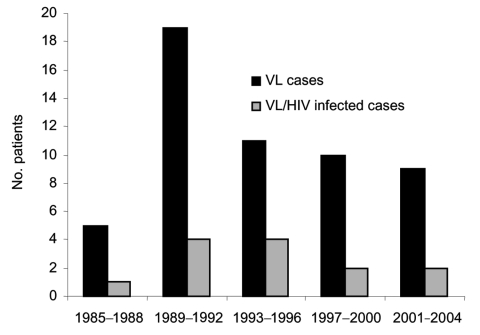
Number of visceral leishmaniasis (VL) cases, United Kingdom, 1985-2004 (data from Health Protection Agency).(data from Health Protection Agency).

UK patients with VL are often referred to the Hospital for Tropical Diseases, London, for confirmation of diagnosis or treatment. We reviewed all cases of VL seen at this hospital from 1985 through 2004. Thirty-nine patients were identified from our hospital database and laboratory records, representing 83% of cases reported in the United Kingdom (Figure 1).

The mean age of these patients was 36 years (range 2–66 years); 4 patients were <15 years of age. The male:female ratio was 2:1. Patients acquired visceral leishmaniasis while in the following areas: 30 (76.9%) in Mediterranean countries (13 in Spain, 9 in Italy, 4 in Greece, 3 in Malta, 1 in Cyprus), 5 (12.8%) from Africa, 3 (7.7%) from Asia, and 1 (2.6%) from South America. During the entire 20-year period, 55.5% of patients had been tourists to these VL-endemic regions, and 44.5% were immigrants or refugees, but after 2000, all patients were tourists. One third of the patients were HIV-antibody positive. Two patients had significant immunosuppression from other causes: 1 from chronic lymphatic leukemia and 1 from immunosuppressive drugs received after kidney transplantation.

Time from onset of symptoms to diagnosis was 1–11 months (mean 3 months). Diagnoses of VL were confirmed by >1 method: microscopic identification of amastigotes in tissue aspirates; histologic examination of biopsy material from bone marrow, liver, or spleen; serologic analysis; and PCR for leishmania DNA (*7*). Leishmania amastigotes were found in 32 bone marrow aspirates, 3 liver biopsy specimens, 2 splenic aspirates, and 1 skin biopsy specimen. PCR to detect leishmania DNA has been performed in this study since 1995 and was positive in 7 of 11 cases. It was the method of confirmatory diagnosis for 1 patient (performed on a bone marrow aspirate that had no visible amastigotes). Serum analysis for leishmania antibodies was performed on samples from 33 patients; results were positive for all HIV-negative patients and for 2 of 13 HIV-positive patients (Table 1).

**Table 1:** Serologic testing in patients with and without HIV infection*

VL cases (no. patients)	IFAT		DAT		LATEX		k39	
	+	–	+	–	+	–	+	–
HIV negative (20)	18	0	20	0	5	0	4	0
HIV infected (13)	1	8	0	9	0	1	1	0

*VL, visceral leishmaniasis; IFAT, indirect immunofluorescent antibody test, 1985–1994; DAT, direct antibody agglutination test (beginning in 1989); LATEX, latex agglutination test, 1996–2003; k39, recombinant k39 antigen detection test (beginning in 2002); +, positive result; –, negative result.

Before 1995, treatment of leishmaniasis involved several drugs, including sodium stibogluconate, paromomycin, meglumine antimoniate, and pentamidine. The latter 2 were used for patients who received initial treatment outside the United Kingdom; within the United Kingdom, most (59%) patients were treated with sodium stibogluconate. After 1995, liposomal amphotericin became the drug of choice and was used in 14 (83%) of 17 patients. HIV status did not affect drug choice.

**Figure 2:**
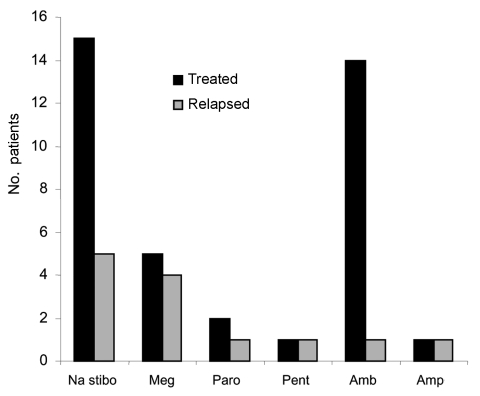
Initial drug treatment for patients with visceral leishmaniasis and number of relapses. Na stibo, stibogluconate; Meg, meglumine antimoniate; Paro, paromomycin; Pent, pentamidine; Amb, ambisome; Amp, amphotericin.

Figure 2 shows the frequency of relapses with each drug treatment in our cohort. Of the 13 patients who had a relapse, 8 (53%) had HIV coinfection. Of the 13 who had HIV coinfection, 8 (62%) had a relapse, compared with 7 (27%) of the 26 HIV-negative patients who had a relapse. Two patients had other risk factors for relapse: 1 had had a kidney transplant, and the other had chronic active hepatitis B. Three patients who had relapses had no apparent risk factors. Relapse occurred in 5 (33%) of 15 patients who received sodium stibogluconate, compared with 1 (7%) of 14 who received liposomal amphotericin as their initial drug. At first, relapsed patients were treated with a combination of sodium stibogluconate and allopurinol, which was unsuccessful in contrast with its reported success in Kenya, or with amphotericin deoxycholate (*8*). HIV-infected patients who had relapses received further treatment with liposomal amphotericin, usually successful, but 2 became unresponsive to treatment. Miltefosine was used in 2 patients with HIV coinfection who had relapses, but relapses recurred, 1 while the patient was still receiving the drug (*9*). Since 2000, pentamidine prophylaxis has been used in 3 immunocompromised patients.

Table 2 summarizes outcomes. The number of patients in the final cure category excludes patients who had had a relapse. The 2 patients who refused treatment had HIV coinfection; 1 died. After 1995, the final cure rate improved and fewer patients required retreatment. The 3 patients who died had advanced HIV disease; 1 had an additional complication of liver failure secondary to hepatitis C cirrhosis.

**Table 2:** Patient response to treatment and outcome

Years	No. patients	Initial cure or response to treatment (%)	Refused treatment (%)	Final cure (%)	Died while receiving treatment (%)
1985–1994	22	7 (35)	2 (9)	18 (90)	2 (9)
1995–2004	17	13 (76)	0	16 (94)	1 (6)

## Conclusions

The data from this cohort show that VL is imported into the United Kingdom at a constant rate, particularly in adult male tourists to the Mediterranean, and that during the past 20 years, HIV and VL had interacted strikingly. The Mediterranean VL-endemic zone accounts for only a small proportion of VL cases globally, but it is a popular holiday destination for the British, and tourism is driving the epidemiology of the imported infection. In the past 5 years, we have seen no immigrants or refugees with VL. The data show that high rates of tourism to an area of low endemicity for a parasitic disease can result in a substantial number of imported cases.

One third of our patients were immunocompromised. The highest rates of reported HIV coinfection worldwide are from Europe, where 85% of the cases are from the southwest; 71% of these are in intravenous drug users (*2*). HIV/VL coinfection is a serious disease that has high death rates, reflected by the 3 deaths in our cohort. These patients had late-stage HIV disease and additional coexisting conditions. HIV/VL-coinfected patients have higher relapse rates and decreased life expectancy. Highly active antiretroviral therapy for HIV is decreasing the numbers of these coinfections and improving survival rates (*10**,**11*).

The mean time from symptom onset to diagnosis was 3 months. This delay resulted mainly from physicians’ failure to consider the diagnosis, although a few cases were difficult to diagnose. For 2 patients, initial microscopic examination of bone marrow biopsy results did not show amastigotes; reinspection showed scarce amastigotes. For another patient, serologic results by direct agglutination were negative when performed early in the course of disease but positive when repeated. The low sensitivity of serologic tests for HIV-infected patients in this cohort confirms that serologic tests cannot be relied on as a means of excluding VL in HIV-infected patients.

Our cohort shows a rise and fall in the proportion of cases of HIV/VL coinfection, starting in the early 1990s, rising to a peak of 50% during 1993–1996, and then decreasing, possibly because of greater use of highly active antiretroviral therapy. Official UK data on VL are incomplete and do not accurately reflect trends. VL remains an imported disease in the United Kingdom and is often associated with HIV infection. We suggest that a formal notification system for visceral leishmaniasis in the United Kingdom, as elsewhere in Europe, would be beneficial for monitoring trends and health planning (*12*). Our data also show the importance of looking at rare imported diseases over a long period so that emerging risk factors can be identified. These data also highlight the usefulness of having 1 center that deals with unusual infections, where expertise in diagnosis and management can be built up and maintained.
